# Elucidating the *in vivo* interactome of HIV-1 RNA by hybridization capture and mass spectrometry

**DOI:** 10.1038/s41598-017-16793-5

**Published:** 2017-12-05

**Authors:** Rachel A. Knoener, Jordan T. Becker, Mark Scalf, Nathan M. Sherer, Lloyd M. Smith

**Affiliations:** 1 Department of Chemistry, University of Wisconsin, Madison, Wisconsin United States; 2 McArdle Laboratory for Cancer Research and Institute for Molecular Virology, University of Wisconsin, Madison, Wisconsin United States; 3 Genome Center of Wisconsin, University of Wisconsin, Madison, Wisconsin 53706 United States

## Abstract

HIV-1 replication requires myriad interactions between cellular proteins and the viral unspliced RNA. These interactions are important in archetypal RNA processes such as transcription and translation as well as for more specialized functions including alternative splicing and packaging of unspliced genomic RNA into virions. We present here a hybridization capture strategy for purification of unspliced full-length HIV RNA-protein complexes preserved *in vivo* by formaldehyde crosslinking, and coupled with mass spectrometry to identify HIV RNA-protein interactors in HIV-1 infected cells. One hundred eighty-nine proteins were identified to interact with unspliced HIV RNA including Rev and Gag/Gag-Pol, 24 host proteins previously shown to bind segments of HIV RNA, and over 90 proteins previously shown to impact HIV replication. Further analysis using siRNA knockdown techniques against several of these proteins revealed significant changes to HIV expression. These results demonstrate the utility of the approach for the discovery of host proteins involved in HIV replication. Additionally, because this strategy only requires availability of 30 nucleotides of the HIV-RNA for hybridization with a capture oligonucleotide, it is readily applicable to any HIV system of interest regardless of cell type, HIV-1 virus strain, or experimental perturbation.

## Introduction

Interactions between the viral unspliced RNAs of human immunodeficiency virus type 1 (HIV) and both virus- and host-encoded proteins play critical roles in viral replication. HIV produces over 40 splice variants from the primary HIV transcript^1,2^, utilizing multiple splice donor and splice acceptor sites. These splice variants fall into three categories: unspliced (~9 kb), partially spliced (~4 kb), and completely spliced (~2 kb). While the completely spliced transcripts pass through the classical nuclear export pathway and are translated into protein products in the cytoplasm, sequence motifs present in the partially spliced and unspliced transcripts lead to the use of alternative cellular pathways to effect both translation and virion assembly^3^. In order to understand these processes, it is critical to identify the proteins that interact with the various forms of the HIV RNAs.

The unspliced HIV transcript is of particular interest because it not only codes for the Gag and Gag-Pol polyproteins, it also serves as the viral genome of the virions that propagate infection. Typically, cellular transcripts that contain introns are either spliced or degraded while in the nucleus. The HIV unspliced transcript, however, interacts with host and viral proteins to prevent its splicing and degradation in the nucleus, promote its nuclear export, and enable its packaging into virions. For example, SR family proteins (proteins with stretches of serine (S) and arginine (R) amino acids) and heterogeneous nuclear ribonucleoproteins (hnRNPs) interact with the splicing enhancers and splicing silencers coded in the transcript to permit alternative splicing^4,5^. Viral protein Rev binds to the Rev Response Element (RRE) of the transcript. This RNA-protein interaction helps the unspliced transcript evade surveillance mechanisms for degradation^4,6^ and facilitates its export by the alternative Rev/CRM1-dependent nuclear export pathway^7^. Furthermore, Rev has been shown to recruit host proteins of diverse functions, including; nuclear matrix protein MATR3^8^, nonsense-mediated decay protein UPF1^9^, and RNA helicases DDX1 and MOV10^10,11^. Following nuclear export, the unspliced transcript can be incorporated into a variety of ribonucleoprotein complexes (RNPs). The binding of host factors such as DDX3 to the highly structured 5′-end of the unspliced transcript has been shown to facilitate translation^12,13^. Comparably, the binding of Staufen1, a double-stranded RNA binding protein, along with HIV-Gag, has been shown to influence the incorporation of the genome into virions^14^. HIV-Gag binds unspliced HIV RNA with sequence specificity via its nucleocapsid domain, leading to encapsidation of the HIV RNA^15^. As the eventual destiny of the unspliced HIV transcript is directly dependent on such interactions with both viral and host proteins, elucidating the identities and roles of these proteins is crucial to understanding the HIV replication process.

Extensive research, using various techniques, has revealed numerous host factors implicated in HIV replication. At least four genomic screens using RNA knockdown techniques to discover critical players in the HIV replication process in cells have been reported^16–19^. The RNA knockdown strategy utilizes small interfering RNAs (siRNAs) or small hairpin RNAs (shRNAs) to knockdown individual genes in cell culture and the effect on HIV infection is monitored^20^. The success of each knockdown requires that the levels of the target transcript and resultant protein are sufficiently decreased in the cells and that the protein product’s function is not replicated by an alternative protein. Despite these limitations, hundreds of genes have been shown in this way to play important roles in HIV replication. However, based on a meta-analysis of three of the four genome-wide RNA knockdown studies, reproducibility of the results is limited and the mechanisms by which most of the proteins act are still not understood^20^. Affinity purification serves as an alternative strategy for identification of pertinent host factors. In this strategy, a protein of interest is tagged with a ligand (e.g. Strep, Flag, GFP), beads coupled to a suitable affinity agent for the ligand are used to capture the desired protein from a complex mixture, and the accompanying proteins are identified by mass spectrometry or other techniques^11,21,22^. A recent example of such a study reported the assembly of viral and host protein networks from the affinity purification of 18 HIV protein products followed by mass spectrometric analysis^22^. A protein affinity purification strategy has also been used to target engineered HIV RNAs in cell lysate and capture associated proteins. Two such studies captured segments of HIV RNA modified with multiple MS2 bacteriophage binding sites, followed by mass spectrometric protein identification^8,23^. Kula *et al*. transfected cells with an HIV-1 derived vector containing truncated HIV RNA sequences modified with 24 MS2 bacteriophage-binding sites. Flag-tagged MS2 coat protein added to the cell lysate binds the MS2 binding sites and the complex is immunoprecipitated using anti-flag antibodies. This strategy revealed many previously known mRNA binders and regulators and also indicated a novel function in HIV replication for MATR3^8^. Similarly, Marchand *et al*. incubated SLS2-A7 HIV-1 RNA segments containing three MS2 binding sites in HeLa cell lysate. The resulting HIV RNPs were then affinity purified using an MS2-maltose binding protein (MBP) fusion protein and amylose beads. This study resulted in the discovery of a novel HIV-1 splicing regulator, hnRNPK. While this RNA-centric affinity purification scheme is valuable for the discovery of RNA-protein interactions, it is limited to use on MS2-containing constructs of HIV RNA, rather than on native HIV RNA sequences.

**Figure 1:**
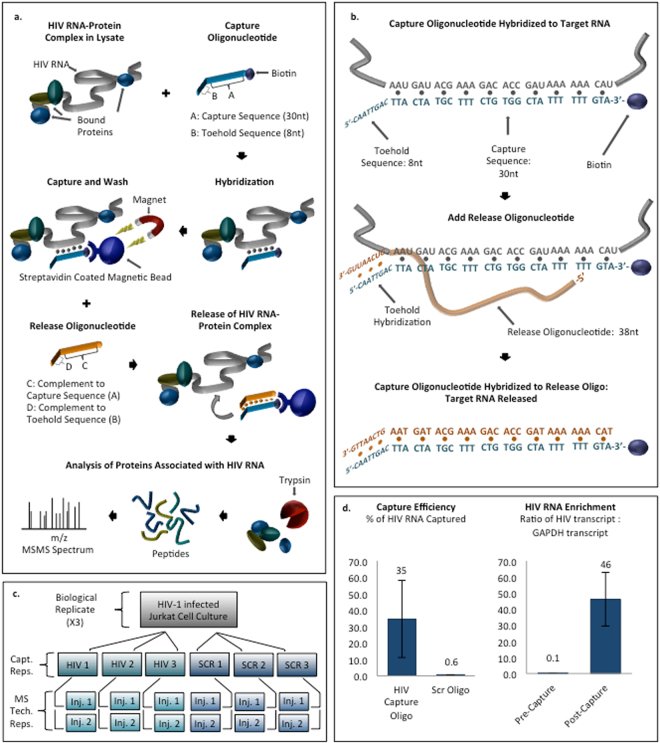
(**a**) HyPR-MS uses hybridization capture for purification of HIV RNA-protein complexes from cell lysate for protein identification by mass spectrometry. (**b**) A toehold-mediated capture and release strategy was implemented for sequence specific isolation of RNA-protein complexes. (**c**) A total of three biological replicates were analyzed. Three capture replicates (Capt. Reps.) were conducted for each biological replicate and were each divided into two mass spectrometric analyses (MS Tech. Reps.). (**d**) Capture efficiency and specificity for each capture was measured using a RT-qPCR assay specific to HIV RNA. RT-qPCR analysis of HIV RNA relative to GAPDH RNA in the capture and lysate samples measured enrichment of HIV RNA after capture. Error bars in graphs are standard deviations of measurements made for all three biological replicates.

Presented here is a versatile strategy for probing *in vivo* HIV RNA-protein interactions we call HyPR-MS: Hybridization Purification of RNA-protein-complexes followed by Mass Spectrometry. HyPR-MS is based upon the specific capture by nucleic acid hybridization of viral RNAs that have been subjected to *in vivo* formaldehyde crosslinking, and mass spectrometric identification of associated proteins. Related strategies have been reported for interrogating gene-specific DNA-protein interactions (PICh: proteomics of isolated chromatin^24^; HyCCAPP: hybridization capture of chromatin associated proteins for proteomics^25,26^) and lncRNA-protein interactions (RAP-MS: RNA antisense purification and mass spectrometry, ChIRP-MS: comprehensive identification of RNA binding proteins by mass spectrometry, CHART-MS: capture hybridization analysis of RNA targets and mass spectrometry)^27–29^. Briefly, the HyPR-MS strategy involves formaldehyde crosslinking of HIV infected cells to preserve RNA-protein interactions. The lysate is then incubated with a biotinylated DNA capture oligonucleotide specifically complementary to the unspliced viral RNA. The capture oligonucleotide hybridizes to the target RNA-protein complexes and the hybrid is isolated from the lysate using streptavidin coated magnetic beads. Following stringent washes the RNA-protein complexes are released from the beads and the sample is analyzed by mass spectrometry to identify associated proteins (Fig. 1a).

HyPR-MS is employed here to identify *in vivo*, unspliced full-length HIV RNA protein interactors. The term “interactors” in this study encompasses proteins that may interact directly with the HIV RNA or proteins that may interact with a protein intermediate that in turn interacts directly with the HIV RNA. Since proteins often function in complexes of multiple proteins, the inclusion of the indirect interactors better elucidates the HIV RNA interactome. The virus encoded RNA binding proteins Rev, Gag/Gag-Pol were among the 189 proteins identified. Additionally, 24 host proteins previously shown to bind to constructs of sequences of HIV RNA and over 90 shown to interact with the RNA-binding viral proteins were identified. This considerable overlap of previously identified HIV-related host factors supports the ability of HyPR-MS to correctly identify RNA-interacting proteins. Many putative novel HIV RNA interactors were also identified including over 25 annotated RNA-binding proteins not previously known to bind HIV RNA. The GO annotations of the proteins identified, as well as the known HIV-related functions of some of the proteins, cover a broad range of RNA-processing steps. Finally, we demonstrate functional effects of several of these proteins using siRNA knockdown techniques followed by fluorescence microscopy to evaluate HIV expression in cells. These findings provide evidence that the HyPR-MS strategy can capture unspliced HIV RNA at various stages of the RNA’s lifespan, and reveal important, functional, *in vivo* HIV RNA protein interactors.

## Results and Discussion

### HyPR-MS overview

Figure 1a shows a diagram of the HyPR-MS strategy. The strategy was applied here to HIV-infected Jurkat cells, a human T lymphocyte cell line that is widely used for the study of HIV infection^30^. Cells were grown in culture, infected with pseudotyped replication-deficient (Env-minus) NL4-3 HIV-1^31^, and crosslinked with formaldehyde to stabilize protein-RNA interactions^32,33^. Cells are lysed with a detergent-containing buffer, briefly sonicated, and cellular debris removed by centrifugation. The cell lysate is then incubated with a biotinylated DNA capture oligonucleotide which contains a 30-nucleotide sequence specifically complementary to the unspliced viral RNA and an 8-nucleotide toehold sequence that is not complementary to the target RNA. The capture oligonucleotide hybridizes to the target RNA-protein complexes and the hybrid is isolated from the lysate using streptavidin-coated magnetic beads. Following stringent washes the RNA-protein complexes are released from the beads using a toehold release oligonucleotide^34^. This oligonucleotide is complementary to all 38 nucleotides of the capture oligonucleotide and is therefore thermodynamically favored to hybridize with it over the target RNA (which is only complementary to 30 nucleotides of the capture oligonucleotide), thereby releasing the HIV RNA-protein complex into solution (Fig. 1b). The sample is then trypsin digested and analyzed by mass spectrometry to identify associated proteins (Details in Supplementary Information and Methods).

### Hybridization capture efficiency and specificity

RT-qPCR assays were employed to measure the yield and specificity of HIV RNA capture (Supplementary Table S1). Control experiments utilizing a scrambled capture oligonucleotide sequence provide a measure of hybridization specificity. The scrambled oligonucleotide was designed to have the same number of nucleotides and approximately the same T_m_ as the capture oligonucleotide but without significant complementarity to the target transcript or any other transcript in the cells (Supplementary Table S2). The hybridization capture strategy was applied to three biological replicates of HIV infected Jurkat cells. A biological replicate is defined here as an independent cell culture and HIV infection followed by cross-linking and pelleting of the cells. Three capture oligonucleotide replicates and 3 scrambled oligonucleotide replicates were produced from each of the three biological replicates to give a total of 9 HIV-RNA oligonucleotide captures and 9 scrambled oligonucleotide captures. The captured and released material from each of these replicates was used mostly for two technical replicates by mass spectrometric analysis for protein identification (Fig. 1c), but a small aliquot (2% of total sample) was saved for verification by RT-qPCR of specificity and efficiency of capture.

Capture efficiency is defined here as a measure of the percentage of the total HIV RNA transcripts in the lysate aliquot that were hybridization captured and released from the beads. It was measured using the HIV RNA-specific qPCR assay to determine the amount of HIV RNA in the capture sample relative to the amount in the lysate prior to capture. The mean capture efficiency for the three biological replicates was approximately 35% (Fig. 1d). Capture specificity is defined here as the amount of HIV RNA in the specific oligonucleotide capture sample compared to that in the scrambled oligonucleotide capture sample. An average of 60-times more HIV RNA was obtained in the HIV capture samples than in the scrambled capture samples (Fig. 1d). We also evaluated the enrichment of the HIV RNA transcript relative to off-target transcripts. Using qPCR assays specific to HIV RNA and to GAPDH RNA (a ubiquitously expressed “housekeeping” gene^35^), we measured the amount of each of these transcripts in both the HIV capture sample and the lysate prior to capture. The ratio of HIV:GAPDH, on average, in the capture sample was 46:1 whereas in the lysate sample the average ratio was 0.13:1. This gives an average enrichment factor of 350 for the three biological replicates (Fig. 1d). These results indicate that the capture procedure provides high specificity and efficiency of HIV transcript capture.

### Mass spectrometry and statistical analysis of the data

The proteins from the HIV capture oligonucleotide and scrambled capture oligonucleotide samples were trypsin digested and analyzed by mass spectrometry. The resulting spectra were searched using MaxQuant^36,37^ for protein identification and label-free relative quantitation^38^ (details in Methods). The Perseus software program^39^ was used to determine which proteins were significantly enriched in the HIV-capture compared to the scrambled-capture samples. In addition to a Student’s T-test p-value, a “student’s T-test test statistic” was calculated with the S_0_ = 0.8. Proteins with a test statistic that pass a permutation-based 1% false discovery rate threshold are considered enriched in the HIV capture samples. The 189 proteins identified in this manner have a maximum p-value of 0.05 and a minimum fold change of 2.2. All 189 p-values are listed as Supplementary Table S3.

### Validation of HyPR-MS protein identifications

HyPR-MS was developed and utilized here in order to identify proteins that interact with unspliced HIV RNA, with a particular interest in those that play roles in HIV replication. Proteins found to interact fall into two major categories: those that have been previously identified as HIV RNA interactors, and those that have not. The former serve to support and validate the effectiveness of the approach, while the latter constitute new discoveries of possible biological significance. To identify previously known interactors, we searched the HIV literature for proteins already shown to (1) interact directly with segments of HIV RNA, or (2) interact with viral proteins (Tat, Rev, Gag, Gag-Pol) that bind to HIV RNA. Two previous studies, Kula *et al*.^8^ and Marchand *et al*.^23^, were particularly valuable resources of known HIV RNA interactors. As described in the Introduction, both of these studies utilized engineered and truncated versions of HIV RNA to permit antibody-based affinity capture; this is in contrast to the HyPR-MS strategy employed here, in which the near-native full-length single-round infectious HIV RNA was captured by hybridization, with *in vivo* RNA-protein interactions stabilized by formaldehyde crosslinking. The study by Kula *et al*. identified 30 HIV RNA-interacting host proteins, while that of Marchand *et al*. yielded 42; 10 and 15 of the Kula and Marchand identifications (with 4 in common), respectively, were found in the present study as well. Additionally, three other proteins have been confirmed in three different studies to interact with HIV RNA^40–42^. In total, 24 host proteins, over 12% of those identified by HyPR-MS, had been previously identified as HIV RNA interactors using orthogonal isolation techniques (Supplementary Table S4).

In addition to the above host proteins, several virus-encoded proteins are known to interact with HIV-RNA: Tat, Rev, Gag, and Gag-Pol as well as Gag-Pol protease products integrase (IN) and reverse transcriptase (RT)^15,43–47^. These proteins enhance HIV-replication by interacting with HIV-RNA at multiple steps during the replication process. Polyproteins Gag and Gag-Pol are translated from the full length HIV-RNA and thus have a large portion of amino acid sequence in common in addition to sections of sequence specific to each polyprotein. HyPR-MS revealed peptides unique to proteins Rev and polyprotein Gag, as well as several peptides that could be from Gag, Gag-Pol or their protein products providing further validation for the technology.

It has been shown that many RNA-binding proteins work in concert with other proteins to perform their functions. The use of formaldehyde as the crosslinking agent in HyPR-MS facilitates the identification of these co-factors because formaldehyde covalently links protein-protein interactors as well as RNA-protein interactors. To this point, it is satisfying that ABCE1 was identified in our study. ABCE1 interacts directly with Gag without an RNA intermediate^48^. The use of formaldehyde crosslinking preserves not just the protein - HIV RNA interaction (in this case Gag – HIV RNA), but also the broader protein-protein interactions (ABCE1 – Gag) of the HIV RNP. Extensive research has been conducted, using various biochemical techniques, to identify proteins that associate with viral-encoded RNA-binding proteins (RBPs) and this data has been compiled into various databases. We cross-referenced our protein list with the NCBI^49^ and GPS-Prot^50^ databases and found that 91 of the 189 proteins identified by HyPR-MS have been previously shown to interact with Tat, Rev, Gag, or Gag-Pol (Supplementary Table S4).

The analysis of the Gene Ontology (GO) annotations^51,52^ for the 189 proteins further confirms the ability of HyPR-MS to identify RNA-associated proteins. Most significantly, 86 proteins are annotated to be RNA-binding. RNA-binding proteins with at least 4-fold enrichment in the HIV capture using HyPR-MS are presented in Table 1. Notably, many of these proteins have been previously shown to interact with HIV-RNA and/or to have functions in HIV replication. Over 90 proteins identified have one or more GO annotations representing involvement in various RNA-related processes. The representation of multiple RNA processes in multiple cellular locations suggests that HyPR-MS is capable of capturing HIV-RNA protein complexes that form throughout the lifespan of the RNA. The GO annotations relating to RNA processing for each protein identified by HyPR-MS are provided in Supplementary Table S5.

**Table 1:** RNA-binding proteins identified to interact with HIV-RNA using HyPR-MS.

Gene ID	Protein Name	Proposed Function in HIV Replication	Previous evidence of HIV-RNA binding?	Refs
**Transcription/Regulation of Transcription**				
FUBP1	Far upstream element-binding protein 1	Unknown	No	
HIST1H1B	Histone H1.5	Unknown	No	
HSPD1	60 kDa heat shock protein	Yes	No	^73^
ILF2	Interleukin enhancer-binding factor 2	Yes	Yes	^23,73,112^
NPM1	Nucleophosmin	Transcription	Yes	^23,73,103^
TRIM28	Transcription intermediary factor 1-beta	Early HIV Replication	Yes	^23,73,90,127^
**Splicing/Splicesome**				
DDX5	DEAD box protein 5	Nucleocytoplasmic Transport	Yes	^23,55,73,126^
EIF4A3	Eukaryotic initiation factor 4A-III	Yes	No	^73^
FUS	RNA-binding protein FUS	Yes	Yes	^8,54^
HNRNPA1	Heterogeneous nuclear ribonucleoprotein A1	Splicing, Nucleocytoplasmic Transport	Yes	^23,61,62,73,116^
HNRNPC	Heterogeneous nuclear ribonucleoprotein C	Yes	No	^73^
HNRNPD	Heterogeneous nuclear ribonucleoprotein D	Splicing, Nucleocytoplasmic Transport	Yes	^23,63,73^
HNRNPK	Heterogeneous nuclear ribonucleoprotein K	Splicing	Yes	^23,65,73^
HNRNPR	Heterogeneous nuclear ribonucleoprotein R	Yes	Yes	^23,65,73^
LUC7L2	Putative RNA-binding protein Luc7-like 2	Yes	No	^73^
PCBP2	Poly(rC)-binding protein 2	Yes	Yes	^42^
RBM8A	RNA-binding protein 8A	Unknown	No	
SYNCRIP	Heterogeneous nuclear ribonucleoprotein Q	Splicing	Yes	^23,73^
**Translation/Regulation of Translation**				
CALR	Calreticulin	Unknown	No	
EEF1A1	Elongation factor 1-alpha 1	Early HIV Replication, Translation, RNA Packaging	Yes	^23,73,111,122^
EEF1D	Elongation factor 1-delta	Early HIV Replication, Translation	No	^73,122^
ELAVL1	ELAV-like protein 1 (HuR)	Translation	No	^70–72^
LRPPRC	Leucine-rich PPR motif-containing protein	Yes	No	^73,120^
NCL	Nucleolin	RNA Packaging/Budding	Yes	^23,73,79^
YBX3	Y-box-binding protein 3	Unknown	Yes	^8^
**P-body/Stress Granule**				
CAPRIN1	Caprin-1	Yes	Yes	^8,73^
MOV10	Putative helicase MOV-10	Nucleocytoplasmic Transport, RNA Packaging	Yes	^8,11,73–76^
PSMA6	Proteasome subunit alpha type-6	Transcription	No	^128^
YBX1	Nuclease-sensitive element-binding protein 1	Yes	Yes	^40,73,92^
**Structural Molecule/Cytoskeleton**				
ANXA1	Annexin A1	Unknown	No	
FLNB	Filamin-B	Unknown	No	
Gag/Gag-Pol	Gag/Gag-Pol polyprotein	Yes	No	^15,45^
RPL10A	60S ribosomal protein L10a	Unknown	No	
RPL11	60S ribosomal protein L11	Unknown	No	
RPL23	60S ribosomal protein L23	Unknown	No	
RPL6	60S ribosomal protein L6	Yes	No	^73^
RPSA	40S ribosomal protein SA	Unknown	No	
SPTBN1	Spectrin beta chain	Unknown	No	^19,105^
TUBA1B	Tubulin alpha-1B chain	Yes	Yes	^8,17,105^
**Other**				
ARF1	ADP-ribosylation factor 1	Yes	No	^102^
CCT6A	T-complex protein 1 subunit zeta	Unknown	No	
HSP90B1	Endoplasmin	Unknown	Yes	^23^
HSPA9	Stress-70 protein	Yes	No	^73^
HSPE1	10 kDa heat shock protein	Unknown	No	
MDH2	Malate dehydrogenase	Unknown	No	
P4HB	Protein disulfide-isomerase	Unknown	No	
PDIA3	Protein disulfide-isomerase A3	Yes	No	^119^
PDIA4	Protein disulfide-isomerase A4	Unknown	No	
PEBP1	Phosphatidylethanolamine-binding protein	Unknown	No	
PPIA	Peptidyl-prolyl cis-trans isomerase A	Early HIV Replication	No	^93,96^
PRDX1	Peroxiredoxin-1	Unknown	No	
PRMT1	Protein arginine N-methyltransferase 1	Unknown	No	
STIP1	Stress-induced-phosphoprotein 1	Unknown	No	
YWHAE	14-3-3 protein epsilon	Unknown	No	
YWHAG	14-3-3 protein gamma	Unknown	No	
YWHAZ	14-3-3 protein zeta/delta	Unknown	No	

**Figure 2:**
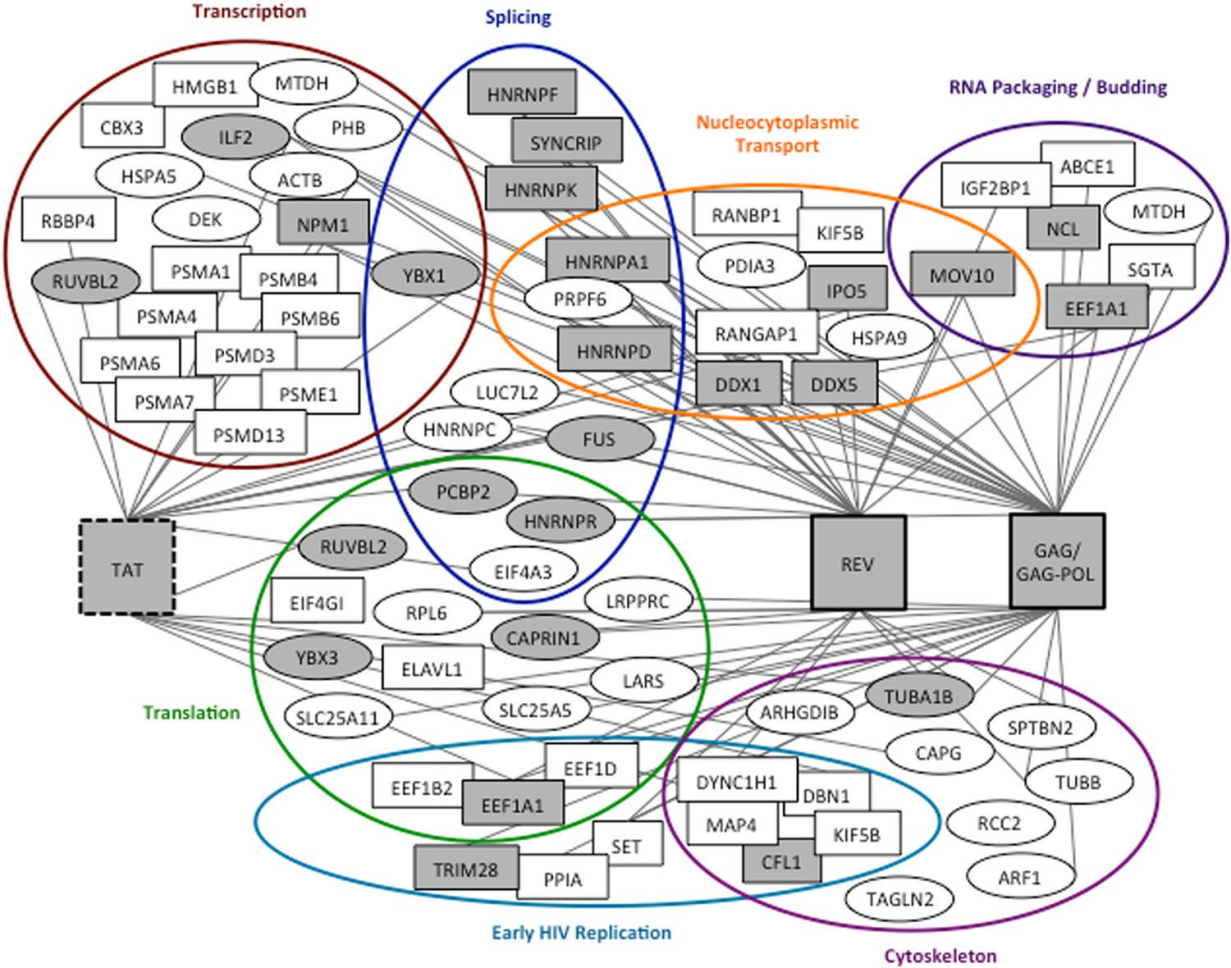
Unspliced HIV RNA protein interactor map. All proteins indicated were identified by HyPR-MS to interact with HIV unspliced RNA (with the exception of Tat). Proteins in rectangles have previously been shown to affect specific stages of HIV replication and are grouped accordingly (colored circles). Proteins in ovals have previously been shown to affect HIV replication but the stage of replication was not determined. These proteins are therefore grouped according to their RNA-process related GO annotation. Proteins shaded in grey have been previously identified to interact with segments of HIV RNA; unshaded proteins are novel unspliced HIV RNA interactors. Virus encoded HIV RNA binders are indicated in the large rectangles; and a line connects them to cellular proteins previously shown to interact with these viral proteins^10,11,16–19,23,40–42,53–56,61–63,65,70–76,79–81,84,90–126^. Supplementary Information Table S3 also lists these interactions.

The proteins identified by HyPR-MS to be unspliced HIV RNA interactors cover a broad range of cellular processes and functions. The list of 189 proteins was investigated for proteins that are known to affect HIV replication in general or at specific stages of the life cycle as well as for proteins that are novel HIV-RNA interactors. Figure 2 shows proteins that were identified in this study to interact with unspliced HIV RNA and were previously shown to affect HIV replication in general (ovals) or at specific stages of the replication cycle (rectangles). The proteins are grouped into RNA-process related categories based on each protein’s known function in HIV replication (rectangles) or, if the specific function is not known, based on the protein’s general GO annotated RNA related functions (ovals). Proteins that have previously been shown to interact with segments of HIV RNA, either *in vivo* or *in vitro*, are indicated with shaded ovals or rectangles. Additionally, lines connect proteins to the virus encoded RBPs (Tat, Rev, Gag, Gag-Pol) that they are known to interact with. This figure is not a comprehensive picture of all proteins identified by HyPR-MS nor of all proteins known to affect HIV replication; however, it provides a summary of perceived significant unspliced HIV RNA protein interactors identified in this study and their relevant interactions and functions. This information is also listed in Supplementary Table S6. Below, we highlight some of these significant and potentially significant HIV RNA interactors.

### HyPR-MS identifies proteins involved in nuclear RNA processes

The unspliced HIV RNA, because it retains its introns, requires mechanisms to prevent its degradation and splicing prior to nuclear export. HyPR-MS identified the viral protein Rev, which is a nuclear export factor that binds to the RRE region of unspliced and partially spliced HIV transcripts and expedites their export into the cytoplasm^6^. In addition to Rev, HyPR-MS also identified RANBP1 and RANGAP1, host factors that are known to be involved in the Rev-dependent nuclear export of RRE-containing RNAs. RANBP1 and RANGAP1 work together to hydrolyze RanGTP to release Rev and its RNA cargo from the export machinery and into the cytoplasm^53^. Interestingly, an *in vitro* study found that PRPF6, another protein also identified by HyPR-MS, interacts with Rev bound to an RRE sequence in an RanGTP dependent manner, suggesting that PRPF6 has a role in HIV RNA nuclear export^54^. The discovery of PRPF6 as an *in vivo* unspliced HIV RNA interactor supports this hypothesis.

Several DEAD-box helicases have been shown to interact with Rev and to impact HIV replication^55^. Two such helicases, DDX1 and DDX5, were identified here as HIV RNA interactors. Previously, co-immunoprecipitation assays from HEK293 cells overexpressing Rev and DDX1 and from 293FT cell extracts expressing Rev and HA-tagged DDX5, confirmed the interaction of Rev with DDX1 and DDX5, respectively^55,56^. Additional studies investigated the impact of these interactions on HIV replication. Both helicases were shown to enhance Rev function using a Rev-dependent luciferase-based reporter plasmid (pDM628) transfected into 293FT cells; DDX5 was suggested to work synergistically with DDX3^55^. Also, siRNA knockdown of DDX1 resulted in restricted Rev function^55,56^ while another study using siRNA knockdown proposed that DDX5 antagonizes the formation of DDX17 homodimers that are required for HIV replication^54^. The study presented here gives a “snap-shot” of *in vivo* RNA-protein and protein-protein interactions, showing that both DDX1 and DDX5 interact either directly or indirectly with HIV RNA. The detection of these interactions was accomplished without modification or overexpression of RNA or protein in the cellular system, providing confidence that the interactions are not artifactual in nature.

Notably, one group of RNA-binding proteins is well represented in the HyPR-MS data: heterogeneous nuclear ribonucleoproteins (hnRNPs). hnRNPs play roles in multiple processes including alternative splicing^57^, mRNA stability^58,59^, and nuclear export^60^. HyPR-MS identified nine hnRNPs; eight (hnRNP A1, D, E2 (PCBP2), F, K, P2 (FUS), Q (SYNCRIP), and R) have previously been shown to bind to select segments of HIV RNA^8,23,42^ and to have roles in HIV replication^61–65^. However, hnRNP C, to our knowledge, has not previously been shown to bind HIV-RNA but has been shown to bind to mRNAs with N^6^-methyladenosine (m^6^A) modifications to affect gene expression^66^. Interestingly, it has been shown recently that HIV-1 RNAs have multiple clusters of m^6^A at the 3′-end and that their presence affects steady state levels of the viral mRNA expression^67^. We further evaluate the effect of HNRNP C on HIV replication using siRNA knock-down techniques described later.

### HyPR-MS identifies proteins involved in cytoplasmic RNA processes

Once it has been exported from the nucleus to the cytoplasm, the unspliced HIV RNA is either translated into Gag and Gag-Pol polyproteins or incorporated into progeny virions. Like cellular mRNAs, viral RNA is assembled into either polysomes for translation or different ribonucleoprotein (RNP) complexes to regulate its translation. Processing bodies (P-bodies) contain mRNAs that are destined for decay whereas stress granules (SGs) contain mRNAs that are stalled in translation initiation^68^. Unspliced HIV RNA can also be assembled into RNPs that are thought to transition the unspliced RNA into progeny virions^14^. These RNPs contain Staufen1 protein and unspliced HIV RNA (thus called SHRNPs) and appear to have a distinct function from other Staufen1 containing RNPs such as stress granules^14^.

Many of the proteins identified by HyPR-MS are multifunctional and are known components of one or more of the above mentioned types of RNPs. Twenty-two proteins with GO annotations in translation, including translation initiation and elongation factors, tRNA ligases, and ribosomal proteins, were identified. Several components of stress granules were also identified, including translation initiation factors, small ribosomal subunit proteins and other translational regulators such as HuR (ELAVL1) and YBX3. HuR has multiple functions in RNA metabolism including regulation of alternative splicing, mRNA stability, and translation^69^. Specific to HIV RNA processing, HuR has been reported to regulate HIV RNA translation^70^; other studies debate whether HuR also influences reverse transcription^71,72^. Like HuR, YBX3 is annotated as a regulator of mRNA stability and translation; however, despite having been shown to bind to HIV RNA constructs^8^ and native unspliced HIV RNA here, its role in HIV replication is still unknown.

Proteins associated with SHRNPs were also well represented in the data presented here. The composition of SHRNPs has been explored using tandem affinity purification and mass spectrometry of Staufen1 containing RNPs from cell lines expressing TAP-Staufen1 and HIV-1^73^. SHRNPs require unspliced HIV RNA for formation and incorporate Gag plus more than 200 cellular proteins^14,73^; 39 of which were also identified by HyPR-MS (Supplementary Table S6). Staufen1, itself, was not identified, which could be due to various experimental factors involving crosslinking, protein abundance, or ionization efficiencies during mass spectrometric analysis. MOV10, a component of P-bodies, is a protein implicated in multiple functions affecting HIV replication including viral infectivity, virion production, reverse transcription, Gag proteolytic processing, and nuclear export^11,74–76^. It has been previously reported that MOV10 binds to engineered constructs of segments of HIV RNA^8^, and now, HyPR-MS shows that MOV10 associates with native unspliced HIV RNA during HIV replication. Additional SHRNP protein components previously shown to affect HIV virion production were also identified by HyPR-MS. ABCE1 has been identified as a necessary factor for HIV-1 capsid formation^77,78^ and NCL has been shown to complex with HIV RNA and Gag to enhance virion release^79^. Conversely, IGF2BP1 has been demonstrated to complex with Gag to block the formation of HIV-1 particles^80^. Protein Lyric (MTDH), though not identified as part of the SHRNP, has been shown to interact with Gag multimers and to be incorporated into HIV-1 virions, suggesting Lyric has an impact on virion infectivity^81^. This hypothesis is further informed by the finding here that Lyric is an unspliced HIV RNA interactor.

### siRNA knockdown and fluorescence microscopy

Eight proteins identified using HyPR-MS (MOV10, YBX3, IGF2BP1, RPSA, HNRNPC, HSPD1, RBBP4, YWHAH) were evaluated for their influence on HIV replication using siRNA knockdown techniques. These proteins were selected for functional assessment for at least one of the following reasons: (1) the p-value comparing HIV capture samples and the scrambled capture samples was <0.05, (2) the total intensities of the peptides for the protein in the HIV capture sample was in the top quartile, or (3) the abundance of the protein in the HIV capture sample was statistically higher than that in a polyA-RNA capture sample (Supplementary Table S7). Details regarding target selection for siRNA knockdown are presented in the Supplementary Information.

**Figure 3:**
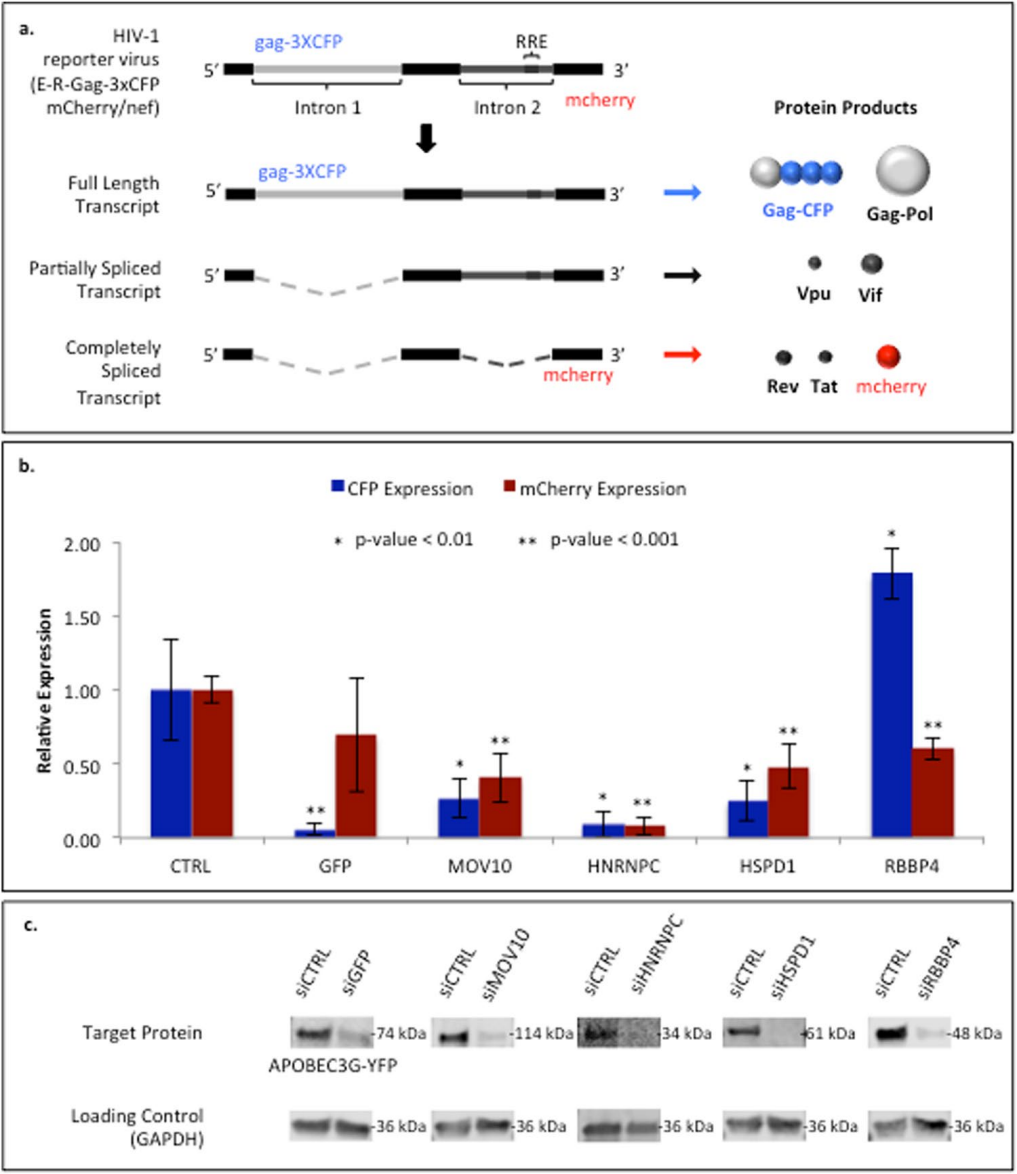
(**a**) Diagram of the HIV-1 reporter virus used for functional evaluation of HIV RNA interactors. The RNA sequences coding for the fluorescent reporter proteins, CFP and mCherry, are postioned so that three CFP and one mCherry molecules are expressed following translation from the full length and the completely spliced transcripts, respectively. (**b**) Quantitative analysis of fluorescence from reporter proteins following target protein siRNA knockdown and 48-hours post HIV infection. (**c**) Western blot analysis of siRNA knocked-down target proteins. Demonstrates >90% knock down of each protein. Images and quantitative analysis of complete blots are in SI Figure S3.

The effect of the knockdown of each target protein was evaluated by, first, transfecting 293T cells (stably-expressing YFP-APOBEC3G) in multi-well culture plates with siRNA pools (Supplementary Table S8) specific for each target protein. In addition to cell cultures transfected with siRNAs against the individual target proteins, two controls were conducted. A sample was transfected with siGFP to knock down the stably expressed cellular YFP-APOBEC3G protein and demonstrate successful transfection conditions. Transfection with non-targeting siRNAs (siCTRL) was used as a negative control. After 48-hours the cells were transfected a second time with the same siRNAs followed by infection with a two-color fluorescent HIV-1 reporter virus (E-R-Gag-3xCFP mCherry/nef; Fig. 3a). This virus is similar to that used for infection of Jurkat cells for HyPR-MS analysis, with two notable changes: the Gag open-reading frame (ORF) contains three copies, in tandem, of CFP reporter and the nef ORF contains an mCherry reporter. Forty-eight hours post-infection the cells were fixed and the nuclei stained (4′,6-diamidino-2-phenylindole, DAPI) and each knockdown-infection sample was imaged using fluorescence microscopy.

After DAPI-staining each multi-well plate was fluorescence-imaged using a Cytation 5 Imaging Reader to quantify cell viability following siRNA transfection. Knockdowns maintaining cell viability within +/− 1.5 standard deviations of the plate mean were considered acceptable (Supplementary Table S9). This resulted in the elimination of RPSA from further evaluation due to excessive cell death following siRNA knockdown.

HIV infection efficiency following siRNA knockdown was assessed using fluorescence microscopy imaging and quantification of CFP and mCherry expression in each sample. The knockdown efficiency of the target proteins was evaluated by western blot analysis showing greater than 87% knockdown of proteins MOV10, HNRNPC, HSPD1, and RBBP4 (Fig. 3c and Supplementary Figure S3). Figure 3b shows the CFP and mCherry intensities, normalized to DAPI-staining, for each of the successful siRNA knockdowns relative to the siCTRL knockdowns. The siGFP sample has a significant decrease in expression of CFP (and not mCherry) further supporting the efficacy of the siRNA knockdown in the samples. All four target proteins knocked down in 293T cells resulted in statistically significant changes in both CFP and mCherry expression upon HIV infection (Fig. 3b and Supplementary Table S10). The knockdown of three proteins (MOV10, HNRNPC, and HSPD1) resulted in a decrease of CFP production while the knockdown of RBBP4 resulted in an increase. Additionally, all four protein knockdowns (MOV10, HNRNPC, HSPD1, and RBBP4) resulted in decreases in mCherry production. The incubation of cells with siRNAs targeting the knockdown of proteins YBX3, IGF2BP1, YWHAH did not result in significant decreases in protein production per western blot analysis and also did not result in statistically significant changes in either CFP or mCherry expression.

We utilized siRNA knockdown of HIV-RNA binding proteins, infection with a dual fluorescence HIV clone (Fig. 3a), and fluorescence microscopy to demonstrate the efficacy of HyPR-MS while revealing potential function of such proteins in the HIV replication process. Four proteins analyzed in this way showed statistically significant changes in CFP and mCherry expression, suggesting their involvement in HIV replication. Of these four proteins, three (MOV10, HNRNPC, and HSPD1) are known RNA-binding proteins (RBPs) (Supplementary Table S5) though only MOV10 has been previously shown to bind to HIV-RNA specifically^8^.

Interestingly, one protein, RBBP4, is not a known RBP; however, it is a known histone binding protein involved in chromatin remodeling, regulation of transcription and negative regulation of gene expression^82,83^. Additionally, RBBP4 has been identified as a component of the HIV pre-integration complex (PIC)^84^ and to interact with the HIV transcription factor Tat (Supplementary Table S4). Our HyPR-MS capture data shows a much higher abundance of RBBP4 in the HIV capture samples relative to the mRNA capture samples (Supplementary Table S7). This suggests that RBBP4 does not interact ubiquitously with mRNAs in general but does interact, either directly or indirectly, with HIV RNA. Based on the above mentioned knowledge of RBBP4 function, it is possible that RBBP4 is not directly binding with the HIV RNA but is interacting through intermediate molecules such as Tat the PIC, or histones at the site of HIV RNA transcription.

The RBBP4 siRNA knockdown data presented here shows an increase in CFP expression and a decrease in mCherry expression; this is a proxy for showing an increase in expression of the full length HIV transcript (CFP) but a decrease in the expression of the completely spliced transcript (mCherry). Though additional experiments would be required to establish the function of RBBP4 in HIV replication, this data suggests that RBBP4 perhaps negatively regulates the expression of the full-length transcript but also could have a role in HIV splicing or in increasing the expression of the completely spliced HIV RNA. Furthermore, the identification of RBBP4 by HyPR-MS as an HIV-RNA interactor demonstrates how the use of formaldehyde crosslinking of RNA-protein complexes allows for the identification of proteins that may not be in direct contact with the RNA but still play a role in HIV replication.

Many HNRNPs have been previously shown to interact with HIV-RNA or protein Rev to affect splicing and/or nuclear export during HIV replication (Supplementary Tables S4 and S6). Many of these were identified here using HyPR-MS. We have also identified an additional HNRNP as an HIV-RNA interactor, HNRNPC; a protein generally involved in RNA stability^58^ and known to bind N6-methyladenosine modified RNAs^66^. The siRNA knockdown of HNRNPC resulted in severe decreases in expression of both CFP (full-length HIV-RNA) and mCherry (completely spliced HIV RNA) supporting that HNRNPC is involved in maintenance of HIV RNA stability.

The knockdown of proteins MOV10 and HSPD1 each show a statistically significant decrease in expression of CFP that is greater than that of mCherry (Fig. 3b). Interestingly, both of these proteins have been previously shown to be components of the Staufen-1 HIV-RNP (SHRNP)^73^. This complex is thought to be, with protein Gag, a conduit for packaging HIV-RNA into virions because the depletion of Staufen1 in infected cells has been shown to reduce Gag protein levels and to deregulate the process of virion formation^14^. The knockdown of the two SHRNP components here results in the decrease of the HIV-RNA that codes for Gag protein, supporting this claim.

### Advantages of HyPR-MS

HyPR-MS identified many proteins with known functions in HIV unspliced RNA processing including alternative splicing, nuclear export, regulation of mRNA stability, translation, regulation of translation, and packaging into virions. This variety suggests that the strategy is capable of capturing unspliced HIV RNA at multiple stages throughout HIV replication. HyPR-MS also identified many proteins that do not yet have known functions in HIV replication, but have GO annotated functions in RNA processing, and in particular RNA-binding. These novel unspliced HIV RNA protein interactors are promising targets for future studies probing host factor functions in HIV replication.

Many techniques are available for interrogating HIV host factors, each unveiling new information about the virus and disease. A subset of such techniques includes those that identify proteins that associate with HIV RNA. These techniques successfully identified proteins from purified sub-genomic or reporter HIV RNAs from transfected, stably-transduced or transformed human cell lines (293T, U2OS, HeLa) using MS2 stem loop/coat protein affinity purification. In contrast, HyPR-MS purified near-native unspliced HIV RNAs from an infected T-cell line (Jurkat) and only relied on the sequence specificity of the capture oligonucleotides to extract RNA-protein complexes of interest. For development of HyPR-MS, multiple experimental iterations were required to optimize the many parameters affecting sample output. It was desirable to have a stable, reproducible, and readily obtained cell type for such studies: for this reason we chose to use the Jurkat T lymphocyte cell line, a widely used model for HIV infection studies^30^. The procedures developed and employed in the present study are quite general, and could readily be adapted to studies on primary cells. The advantage of HyPR-MS is threefold: (1) the strategy does not require modification or overexpression of the HIV RNA nor any cellular proteins giving confidence that the interactions are not artifactual in nature; (2) the RNA-protein interactions are preserved *in vivo* by formaldehyde crosslinking prior to any perturbation of the cellular system giving confidence that the interactions are biologically relevant; and (3) only 30 nucleotides of the native HIV RNA sequence is required for purification of the complexes permitting the application of HyPR-MS nearly universally with regards to HIV-1 strain, cell line and cell type. The versatility of the HyPR-MS technique as well as its ability to capture unspliced HIV RNA throughout its lifespan suggests it will be a powerful tool for interrogation of *in vivo* HIV RNA-protein interactions under various experimental perturbations to gain insights into how HIV replication occurs and can be prevented. Although the focus of this study is the interactions of proteins with HIV RNAs, the technology itself is likely to be readily adapted to other RNA species such as mRNAs and lncRNAs. In fact, preliminary work in our laboratory applying HyPR-MS to both types of RNA targets is underway and will be presented in future publications.

## Methods

### Cell Culture, Virus Production, and Infections

HEK293T cells were cultured in DMEM media supplemented with 10% fetal bovine serum and 1% L-glutamine-penicillin-streptomycin at 37ºC in 5% CO_2_. To generate single-round infectious HIV-1 virions, 2.5X10^6^ HEK293T cells were plated in 10 cm tissue-culture treated dishes in 10 mL media. Each 10 cm dish was transfected using polyethylenimine (PEI) with 1 µg of DNA plasmid expressing the G envelope glycoprotein from vesicular stomatitis virus (VSV-G) and 9 µg of plasmid DNA encoding the full-length NL4-3 molecular clone of HIV-1 bearing inactivating mutations in *env*, *vpr*, and expressing a Cyan Fluorescent Protein (CFP) reporter from the *nef* reading frame (HIV-1 E-R-CFP)^85–87^. At 24 hours post-transfection, old media was removed and replaced with 4 mL fresh media. At 48 hours post-transfection, culture supernatants were harvested, filtered through a sterile 0.45 µm syringe filter, aliquoted, and frozen at −80 °C. Infectivity of virus produced was determined by small-scale infection of Jurkat T-cells to quantify dose of viral inoculum required for large-scale infections. Jurkat T-cells (obtained from ATCC) were cultured in RPMI media supplemented with 10% fetal bovine serum and 1% L-glutamine-penicillin-streptomycin. Jurkat T-cells were expanded in 850 cm^2^ tissue culture treated roller bottles rotated at 3 rotations per minute. A cell density of 1X10^6^ cells per mL of media was maintained by regular quantification. 300X10^6^ Jurkat T-cells were infected in a low volume (typically 25 mL RPMI + 25 mL viral inoculum in DMEM) for three hours rotating as above. Polybrene was added at a concentration of 10 µg/mL to increase infectivity. After three hours, culture volume was increased to 300 mL using RPMI media. Infected cells were incubated and rotated as above for 45 hours. Successful infection was confirmed by visualizing CFP expression via epifluorescent microscopy. Typically >90% of cells were infected. Uninfected control cells were treated similarly (combined with 25 mL DMEM media instead of viral inoculum). At this point, cells were concentrated via centrifugation at 1500 rpm for 10 minutes and washed three times with PBS. Washed cells were cross-linked by resuspending in 0.25% formaldehyde (diluted in PBS) and incubated at room temperature for 10 minutes prior to centrifugation. Cross-linked cells were washed once with 1xPBS and then resuspended in 100 mM Tris-HCl (pH 7.4) to quench cross-linking reactions for 10 minutes at room temperature. Cells were washed twice more in 1xPBS, pelleted by centrifugation, and frozen at −80ºC.

### Special Considerations for HyPR-MS

All solutions used for cell lysis, hybridization, capture, release, and RT-qPCR were prepared using certified RNase free components. Water added to all solutions was nuclease free UltraPure distilled water (Invitrogen, 10977-015).

The amount of lysate, the concentration of capture-oligonucleotides, and the volume of streptavidin coated magnetic beads needed in each capture experiment for identification of proteins interacting with HIV RNA is conditional on the number of copies of the HIV RNA present in the infected cell culture. The capture oligonucleotide concentration and volume of streptavidin coated magnetic beads needed for optimal capture efficiency and specificity for each biological replicate was empirically determined. To do this, small-scale capture experiments using 5X10^5^ cells were performed using increasing amounts of capture oligonucleotides and streptavidin coated beads. The amount of HIV RNA captured was then measured using RT-qPCR (as described below) and the oligonucleotide concentration and bead volume producing the highest capture efficiency while maintaining a desirable capture specificity was scaled up for the large-scale capture experiments. The number of cells needed for a capture experiment for protein identification was then estimated and empirically confirmed by mass spectrometry. For the data presented here, 7.5X10^7^ HIV-1 infected Jurkat cells, 188 pmol of capture or scrambled oligonucleotides, 1.125 mL of streptavidin coated beads, and 188 nmol of release oligonucleotides were used for each capture sample.

### Cell Lysis

Cells were resuspended on ice in lysis buffer (469 mM LiCl, 62.5 mM Tris HCl, pH 7.5, 1.25% LiDS, 1.25% Triton X-100, 12.5 mM Ribonucleoside Vanadyl Complex, 12.5 mM DTT, 125 U/mL RNasin Plus, 1.25X Halt Protease Inhibitors) to a final cell concentration of 5X10^6^ cells/mL. Cells were lysed by frequent vortexing for 10 minutes, keeping the cells on ice between vortexes. The cell lysate was then sonicated on ice for 30 seconds with 4 seconds of rest between each 4-second sonication interval. The lysate was then centrifuged at 1000 g for 2 minutes at 4 °C and the supernatant transferred to a new tube.

### Hybridization and Capture

The lysate was diluted with nuclease free water so that the final component concentrations for hybridization are as follows: 375 mM LiCl, 50 mM Tris, 1% LiDS, 1% Triton X-100, 10 mM RVC, 10 mM DTT, 100 U/mL RNasin Plus, 1X Halt Protease Inhibitors. The HIV-capture oligonucleotide or the scrambled oligonucleotide (Table S2) was added to 15 mL of diluted lysate and the sample was incubated while nutating at 37 °C for 3 hours. The predetermined volume of streptavidin coated magnetic Speedbeads (Thermo Fisher Scientific, 09981140) was washed 3 times with and resuspended in three volumes of bead wash buffer (375 mM LiCl, 50 mM Tris, 1% LiDS, 1% Triton X-100) prior to addition to each hybridization mixture. The bead capture mixture was then nutated at 37 °C for 1 hour. Following incubation the beads were collected to the side of the tube using a magnet and the remaining lysate was removed. A volume of pre-warmed wash buffer (100 mM LiCl, 50 mM Tris, 0.2% LiDS, 0.2% Triton X-100, 37C) that was 5X the volume of beads used for capture in the sample was used to resuspend the beads and the beads were washed at 37 °C for 15 minutes. The beads were then washed with a 5X volume of release buffer (375 mM LiCL, 50 mM Tris) at room temperature for 5 minutes.

### Release from Beads

The beads were resuspended in release buffer (a volume 3X that of the beads used for capture) and the release oligonucleotide (Table S2) was added. The mixure was nutated at room temperature for 30 minutes followed by magnetic separation of the beads from the supernatant containing the released RNA-protein complexes. The solution was transferred to a new tube and divided into aliquots for RT-qPCR and mass spectrometric protein analysis.

### RT-qPCR

Samples were incubated at 37 °C with 1 mg/mL proteinase, 4 mM CaCl_2_, and 0.2% LiDS. The RNA was extracted with TriReagent (Sigma, T9424) per manufacturer’s protocol and was then precipitated in 75% ethanol at −20 °C for several hours. The tube was then centrifuged at 20,800 g to pellet the RNA and the RNA was then washed with 75% ethanol. The RNA pellet was resuspended in 15 uL of nuclease free water and 10 uL was used for reverse transcription (High Capacity cDNA Reverse Transcription Kit, Applied Biosystems) per the manufacturer’s protocol. The resulting cDNA sample was analyzed with sequence-specific qPCR assays (Table S1) for quantitation of relevant transcripts.

### eFASP

The protocol for protein preparation was adapted as follows from that described by Erde, J. *et al*.^88^. Amicon 50 kDa MWCO filters (Millipore, UFC505096) and collection tubes were passivated by incubating overnight in 1% CHAPS and then rinsed thoroughly with mass spectrometry grade water. Each release sample was brought to 0.1% deoxycholic acid and 8 M urea. The sample was passed through the filter in 500 uL increments by centrifugation for 10 minutes at 14,000 g and the eluant was discarded. 400 uL of exchange buffer (8 M urea, 0.1% DCA, 50 mM Tris pH 7.5) was added to the filter and the tube was centrifuged at 14,000 g for 10 minutes. This was repeated for a total of 3 exchanges. 200 uL of reducing buffer (8M urea, 20 mM DTT) was added to the filter and the sample was incubated for 20 minutes at room temperature followed by centrifugation. 200 uL of alkylation buffer (8 M urea, 50 mM iodoacetamide, 50 mM ammonium bicarbonate) was then added to the sample, followed by incubation for 20 minutes at room temperature in the dark, and centrifugation at 14,000 g for 10 minutes. Finally, the sample was exchanged with 3 aliquots of 400 uL of digestion buffer (1 M urea, 50 mM ammonium bicarbonate, 0.1% DCA) and resuspended in a final volume of 100 uL of digestion buffer. Trypsin was added to the sample, the filter was transferred to a fresh, passivated collection tube, and the cap was sealed with parafilm followed by incubation overnight at 37 °C for protein digestion. The filter-collection tube was centrifuged for 10 minutes at 14,000 g. 50 uL of 50 mM ammonium bicarbonate was added to the filter and centrifuged at 14,000 g for 10 minutes. This step was repeated once to ensure the collection of the entire peptide sample. The 200 uL peptide sample was then brought to 1% TFA followed by addition of 200 uL of ethyl acetate. The sample was vortexed for 1 minute then centrifuged at 15,800 g for 2 minutes. The top layer was aspirated and discarded and extraction with ethyl acetate was repeated 2 times. The aqueous layer was then dried using a Savant SVC-100H SpeedVac Concentrator and the sample resuspended in 150 uL 0.1% TFA. For removal of salts from the sample a C18 solid-phase extraction pipette tip (OMIX C18, 100 uL, Agilent Technologies) was first conditioned with 70% ACN, 0.1% TFA, and then equilibrated with 0.1% TFA. The peptide sample was then loaded onto the C18 solid phase by repeated passing of the 150 uL sample over the cartridge. The OMIX pipette tip was then rinsed with 0.1% TFA 10 times followed by peptide elution in 150 μL 70% ACN, 0.1% TFA. The samples were then dried using the SpeedVac Concentrator and reconstituted in 95:5 H2O:ACN, 0.1% formic acid.

### Mass Spectrometry of Peptides

The samples were analyzed using an HPLC-ESI-MS/MS system consisting of a high performance liquid chromatograph (nanoAcquity, Waters) set in line with an electrospray ionization (ESI) Orbitrap mass spectrometer (LTQ Velos, ThermoFisher Scientific). A 100 μm id X 365 μm od fused silica capillary micro-column packed with 20 cm of 1.7 μm-diameter, 130 Angstrom pore size, C18 beads (Waters BEH) and an emitter tip pulled to approximately 1 μm using a laser puller (Sutter Instruments) was used for HPLC separation of peptides. Peptides were loaded on-column with 2% acetonitrile in 0.1% formic acid at a flow-rate of 400 nL/minute for 30 minutes. Peptides were then eluted at a flow-rate of 300 nL/minute over 120 min with a gradient from 2% to 30% acetonitrile, in 0.1% formic acid. Full-mass profile scans (300–1500 m/z) were performed in the FT orbitrap at a resolution of 60,000. The ten highest intensity parent ions were selected for MS/MS HCD scans at 42% relative collision energy, 7,500 resolution, and with a mass range starting at 100 m/z. Dynamic exclusion was enabled with a repeat count of two over a duration of 30 seconds and an exclusion window of 120 seconds. The Orbitrap raw files were analyzed using MaxQuant (version 1.5.3.30)^36^ and searched with Andromeda^37^ using the combined Uniprot^51^ canonical protein databases for human and HIV-1 and supplemented with common contaminants (downloaded June 8, 2016). Samples were searched allowing for a fragment ion mass tolerance of 20 ppm and cysteine carbamidomethylation (static) and methionine oxidation (variable). A 1% false discovery rate for both peptides and proteins was applied. Up to two missed cleavages per peptide were allowed and at least two peptides were required for protein identification and quantitation. Protein quantitation was achieved using the sum of the peptide peak intensities for each protein of each biological replicate and capture sample type. The peak intensities of HIV capture samples were normalized by the total peak intensity of all HIV capture samples and the same was done for scrambled capture samples.

### Statistical Analysis of Protein Data

The intensities for each protein were then analyzed using the Perseus^39^ companion software to MaxQuant for statistical differences between the 3 HIV capture and 3 scrambled capture biological replicates. Following log transformation of peak intensities, missing values were imputed with the width setting at 0.3 and the downshift set to 1.8. A permutation-based 1% FDR analysis of these values, with S_0_ set to 0.8, produced 189 proteins statistically enriched in the HIV capture samples compared to the scrambled capture samples.

### Cell culture, stable cell line, and HIV-1 reporter virus

Human 293T cells stably-expressing YFP-APOBEC3G were cultured in Dulbecco’s modified Eagle’s medium (DMEM) supplemented with 10% fetal bovine serum, 1% L-glutamine, and 1% penicillin-streptomycin. For all experiments, cells were maintained at 37 °C and 5% CO_2_ in a humidified incubator. The virus used was a two-color fluorescent HIV-1 reporter virus (E-R- Gag-3xCFP mCherry/nef) which expresses mCherry in the nef ORF and three copies of CFP, in tandem, between the matrix and capsid ORFs of Gag, in a similar but expanded manner as Mergener 1992, Muller 2004, Holmes 2015, and Hendrix 2015. This virus allows screening for early (mCherry; multiply-spliced genes) and late (CFP; unspliced gene products). Stocks of viral inoculum were produced in 293T cells by transfecting the E-R- Gag-3xCFP mCherry/nef with psPAX2 and VSV-G.

### siRNA knockdown and infection

For siRNA knockdown cells were cultured in 48-well plates. A dilution of DarmaFECT transfection reagent in Opti-MEM was mixed with the appropriate siRNA pool also diluted in Opti-MEM and allowed to incubate for 20 minutes. This solution was then brought to 300 uL with antibiotic free DMEM, mixed well, and used to replace the existing media in each well. The final, in-well concentration of each siRNA was 25 nM. After four hours of incubation, the media was replaced with fresh media and the cells were incubated overnight. Approximately 24-hours post transfection the cells were split into two plates and a second siRNA transfection as described above was conducted an additional 24-hours later, in both plates. Again, four hours post transfection the siRNA containing media was replaced with fresh media. Additionally, in one plate, polybrene was added to the media followed by the HIV-1 reporter virus (E-R- Gag-3xCFP mCherry/nef). After 24 hours the media is exchanged for fresh media and 48-hours post infection the cells are washed with PBS and fixed for 12 minutes using 4% paraformaldehyde (PFA) in PBS then stored at 4C in PBS until imaged.

### DAPI staining and fluorescence imaging

Fixed cells were permeabilized using 0.2% Triton X-100, and stained with DAPI (4′,6′-diamidino-2-phenylindole 4′,6-diamidino-2-phenylindole). DAPI fluorescence was measured using a Cytation 5 Imaging Reader (Biotek Instruments, Inc.) operated by Gen5 software (v 2.07) using excitation/emission monochromator range (wavelengths in nanometers) 340 to 380/420 to 480 (DAPI). Cell number based on the relative DAPI signal is later used to normalize imaging data. Additional imaging experiments were performed on a Nikon Ti-Eclipse inverted wide-field epifluorescent deconvolution microscope (Nikon Corporation). Images were collected using an Orca-Flash 4.0 C11440 (Hamamatsu Photonics) camera and Nikon NIS Elements software (v 4.20.03) using Nikon 4x/0.13 (Plan Apo) objective lense and the following excitation/emission filter set ranges (wavelengths in nanometers): 418 to 442/458 to 482 (CFP), 490 to 510/520 to 550 (YFP), 555 to 589/602 to 662 (mCherry). Images were processed and analyzed using FIJI/ImageJ2^89^. Results were obtained from two biological replicates, defined as cells treated with siRNAs and infected with virus on separate days.

### Western blot analysis

For western blot analysis cells were prepared identically to those described in “siRNA knockdown and infection” except instead of paraformaldehyde fixation the cells were lysed in radioimmunoprecipitation assay (RIPA) buffer (10 mM Tris-HCl [pH 7.5], 150 mM NaCl, 1 mMEDTA, 0.1% SDS, 1% Triton X-100, 1% sodium deoxycholate) containing complete protease inhibitor cocktail (Roche). The cell lysates were then sonicated briefly and centrifuged for 10 minutes at 1000xg then boiled in dissociation buffer (62.5 mM Tris-HCl [pH 6.8], 10% glycerol, 2% sodium dodecyl sulfate [SDS], 10% beta-mercaptoethanol) at a 1:1 ratio. SDS-PAGE was performed on a 4% to 15% polyacrylamide gel, and the proteins were transferred onto a nitrocellulose membrane. The membranes were incubated with the appropriate antibodies (Supplementary Table S8) at a 1:1000 dilution overnight. Additionally, the gene encoding GAPDH, a housekeeping gene, was detected with an additional antibody in each membrane-incubation to normalize band density values to total cell protein values. Following washing of the membranes, secondary IRdye680- or IRdye800-conjugated antibodies (Supplementary Table S8) were incubated at 1:10000 dilution for 1.5 hours and used for quantitative immunoblotting with an Odyssey infrared scanner (Li-Cor Biosciences).

### Data Availability

The corresponding author may be contacted for questions regarding all data and protocols. The MS datasets generated during the current study are available in the MassIVE repository (https://massive.ucsd.edu/ProteoSAFe/static/massive.jsp).

## Electronic supplementary material

Supplementary Information

Supplementary Tables
