# Supplementary material for: Elucidating the in vivo interactome of HIV-1 RNA by hybridization capture and mass spectrometry
Source: Sci Rep. 2017 Dec 5;7:16965. doi: 10.1038/s41598-017-16793-5 (PMC5717263; doi:10.1038/s41598-017-16793-5)
Supplement: Supplementary file 1 — Supplementary Information [file 41598_2017_16793_MOESM1_ESM.doc]

**Elucidating the *in vivo* interactome of HIV-1 RNA by hybridization capture and mass spectrometry.**

Rachel A. Knoener^1^, Jordan T. Becker^2^, Mark Scalf^1^, Nathan M. Sherer^2^, Lloyd M. Smith^*1,3^

^1^Department of Chemistry, University of Wisconsin, Madison, Wisconsin, United States

^2^McArdle Laboratory for Cancer Research and Institute for Molecular Virology, University of Wisconsin, Madison, Wisconsin, United States

^3^Genome Center of Wisconsin, University of Wisconsin, Madison, Wisconsin 53706, United States

^*^Corresponding Author:

Lloyd M. Smith; smith@chem.wisc.edu

SUPPLEMENTARY INFORMATION

The objective of HIV-HyPR-MS is to use hybridization capture of unspliced HIV RNA-protein complexes to identify proteins relevant to the process of HIV replication. Several challenges must be addressed to succeed in this goal.

1. Biological Relevance: *In vivo* RNA-protein interactions must be stabilized to withstand downstream experimental conditions.
2. Specificity of Capture: Unspliced HIV RNA-protein complexes must be specifically isolated from a complex lysate milieu.
3. Magnitude of Capture: High capture efficiency must be achieved so that a reasonable number of cells can be used for identification of proteins by mass spectrometry.
4. Determination of “true binders”: Proteins that truly interact with the HIV RNA (true binders) must be differentiated from non-specific interactors using a combination of control experiments and statistical analysis strategies.

Below we describe the experimental strategies employed in HyPR-MS to overcome these challenges.

**Culture, HIV-1 Infection, and Crosslinking of Jurkat T-cells.**

Cell type selection and the HIV infection strategy are integral factors for obtaining both a sufficient magnitude of capture and biological relevance. Jurkat T-cells were selected because they are amenable to growth in large numbers as compared to primary peripheral blood mononuclear cells (PBMC) or purified CD4+ T-cells. Additionally, Jurkat T-cells largely recapitulate the cellular environment that HIV typically infects (CD4+ T-cells) ensuring that the hybridization capture results will be biologically relevant. HIV-1 infection was achieved by using the replication incompetent NL4-3 molecular clone. The use of a molecular clone allowed us to know the genomic sequence, gene expression, and kinetics of the viral life cycle while also maintaining experimental reproducibility. Because the development of this multistep, complex technology requires several experimental iterations to establish the parameters optimal for success, Jurkat T-cells infected with the NL4-3 HIV molecular clone were ideal selections.

RNA-protein interactions in the cell are often dynamic, transient, and labile. These interactions must be stabilized so that biologically relevant interactions are preserved while non-specific interactions are disrupted during the HyPR-MS process. To achieve this the HIV infected cell culture is incubated with formaldehyde. Formaldehyde is a fast acting, zero-length, membrane permeable covalent cross-linker that only crosslinks molecules that are in contact with each other. Once covalently linked, RNA-protein and protein-protein interactions are preserved while non-specific interactions are disrupted using harsh salt and detergent concentrations experimentally downstream. This helps to ensure that proteins identified are either directly interacting with HIV RNA or are interacting with other proteins that directly interact with HIV RNA.

**HIV Capture Oligonucleotide and Scrambled Capture Oligonucleotide Design and Placement.** The capture oligonucleotide is a key design feature of HyPR-MS for obtaining specificity of capture. The oligonucleotide contains three elements: a 30-nucleotide (nt) complementary sequence, a 3’-biotin, and a 5’ 8-nt non-complementary sequence (toehold). The complementary sequence is designed to specifically hybridize to the unspliced HIV RNA, the biotin then anchors the target RNA-protein complex to the streptavidin coated magnetic beads, and the toehold sequence provides a mechanism for release of the HIV RNA-protein complex from the beads. The capture oligonucleotide must be designed so that it hybridizes to the unspliced HIV RNA but does not hybridize to any other transcript in the lysate, including the partially and completely spliced HIV RNA variants. The complementary sequence of the oligonucleotide was designed to be complementary to a region in a ~5kb sequence that is retained in the unspliced transcript but is spliced out of both the partially and the completely spliced transcripts (Supplementary Figure S1). The region of hybridization was further informed by the secondary structure of the full length HIV RNA. Watts, J, et. al. used Selective 2′-Hydroxyl Acylation Analyzed by Primer Extension, or SHAPE, to determine the structure of the HIV-1 genome in virions. SHAPE determines the probability that a given nucleotide is not base-paired, or is single stranded. Based on this information, the capture oligonucleotide was designed to be complementary to a region that has a high probability to be single stranded and thus more likely to be accessible to the capture oligonucleotide. Finally, an online Basic Local Alignment Search Tool (BLAST) was used to determine that the capture oligonucleotide sequence is sufficiently specific to the target transcript so that wash conditions remove any off-target interactions. The complementary sequence of the capture oligonucleotide is a 30-nt DNA sequence with a T_m_ of ~68ºC at the conditions of hybridization (Supplementary Table S2). Following hybridization of the capture oligonucleotide to the HIV RNA, the streptavidin coated magnetic beads are added to the lysate. The biotin of the capture oligonucleotide then anchors the HIV RNA-protein complex to the beads and a magnet is used to pull the beads to the side of the tube so that the remaining lysate can be removed and the beads can be washed.

The capture oligonucleotide design incorporates a mechanism for release of the HIV RNA-protein complexes from the beads. We have developed a strategy that incorporates an 8-nt toehold sequence in the capture oligonucleotide for a programmable release from the beads. The beads are resuspended in a release buffer and the 38-nt toehold release oligonucleotides are added to the solution. The release oligonucleotide is designed to be fully complementary to the entire 38-nt capture oligonucleotide. The 8-nt sequence on the capture oligonucleotide, which is not complementary to the target RNA, hybridizes with its complement on the release oligonucleotide. This gives the release oligonucleotide a “toehold” to hybridize with the remaining of the capture oligonucleotide. Since the target RNA is only complementary to 30-nt of the capture oligonucleotide and the release oligonucleotide is fully complementary, the interaction of the capture oligonucleotide with the release oligonucleotide is thermodynamically more favorable and the target RNA is released from the capture oligonucleotide into solution.

A second oligonucleotide, the scrambled oligonucleotide, was also designed to serve as a negative control capture. This scrambled oligonucleotide was designed to have the same number of nucleotides and approximately the same T_m_ as the capture oligonucleotide but does not have significant complementarity to the target transcript nor any other transcript in the cells (Supplementary Table S2). Capture experiments using the capture oligonucleotide and the scrambled oligonucleotide were performed in parallel.

**HIV RNA Quantitation.** Each capture sample was analyzed using RT-qPCR to determine the effectiveness of our technology for purifying HIV RNA-protein complexes. Determination of the capture efficiency and specificity and enrichment of HIV was achieved using qPCR assays specific to HIV RNA and to GAPDH RNA (Supplementary Table S1). Since unspliced HIV RNA is reverse transcribed in the host cells during HIV replication, there is potential to capture the HIV DNA in addition to the HIV RNA. To ensure that the nucleic acid captured by HyPR-MS is, in fact, RNA, we analyzed the capture samples by RT-qPCR without reverse transcriptase added to the reverse transcription reactions. For these experiments, it should be noted that, the RNA/DNA purification step used a phenol-chloroform extraction instead of the usual Trizol extraction so that both RNA and DNA captured would be isolated from the capture sample. The polymerase used for qPCR does not bind and amplify RNA so any signal obtained in the no-reverse transcriptase reactions will be due to the presence of DNA. From this analysis it was determined that the presence of DNA is over 3 orders of magnitude lower than RNA in the capture samples ensuring proteins detected are associated with HIV RNA, not DNA (Figure S2).

**Protein target selection for functional analysis.** The statistical analysis used to identify HIV-RNA protein interactors (student’s t-test with 1% permutation-based FDR) identified about 190 potential proteins for functional analysis. We sought to select several of these for analysis using three parameters: the p-value obtained from the student’s t-test analyzing HIV capture compared to scrambled capture; the intensity or abundance of the protein in the HIV capture samples; and specificity of the HIV RNA-protein interaction by determining if that interaction is common among other cellular RNAs or if it is relatively unique to the HIV-RNA. First, the p-value parameter is obtained from the statistical analysis described above and is presented in Supplementary Table S3 and again in Supplementary Table S7 for this analysis. The primary purpose of this statistical test here is to detect differences in protein quantities between the HIV capture samples and the negative control (scrambled capture samples). This test accounts for the variability among biological replicates for determining significant protein quantity differences between the HIV and scrambled capture samples. The second parameter seeks to eliminate proteins that might be present in the HIV capture samples in low abundances that may be near the limit of quantitation of the MS. Using peak intensities for determining relative peptide abundance between samples is a powerful tool that permits relative quantitation without laborious and expensive labeling techniques such as SILAC and TMT-tagging. Any quantitative method has a lower limit of quantitation that is typically determined using a calibration curve. However, each peptide varies in its properties, such as ionization efficiency, which would require that a calibration curve be implemented for each individual peptide to determine that peptide’s limit of quantitation. This is not feasible since there are thousands of peptides in each sample. We sought to avoid selecting proteins with low abundances that may be near the limit of detection and thus have more uncertainty in their calculations. Therefore, for the purpose of selecting proteins for functional analysis, we calculated the sum of a protein’s peptide intensities in the HIV capture samples and flagged proteins with a total protein intensity in the top quartile of total intensities for all proteins. Lastly, we sought to select for proteins that are not ubiquitous binders to mRNA but are somewhat selective for HIV RNA specifically. To do this we performed a HyPR-MS capture experiment using poly-dT capture oligonucleotides that hybridized with the common poly-A tail found on mature mRNAs. We determined the p-value using the student’s t-test for proteins in the HIV capture samples and the total mRNA capture samples as well as the fold difference in protein intensity between the two sample types. These values are also presented in Supplementary Table S7. These three parameters combined with understanding from the literature of the known general function of the protein and its specific function with regards to HIV replication informed our selection of the proteins for functional analysis.

**Western Blot Analysis:** The blots shown in the main text are only cut-outs of the pertinent proteins targeted for siRNA knockdown. Figure S3 shows the full blot for each siRNA knockdown/western blot along with the ladder for indication of the protein sizes. Quantitation was done using ImageStudioLite to compare the intensity of fluorescence for the knock-down protein and the loading control protein for each western blot. The percentage of knockdown for each targeted protein was determined using these numbers and normalizing using the values obtained for GAPDH.

SUPPLEMENTARY FIGURE LEGENDS


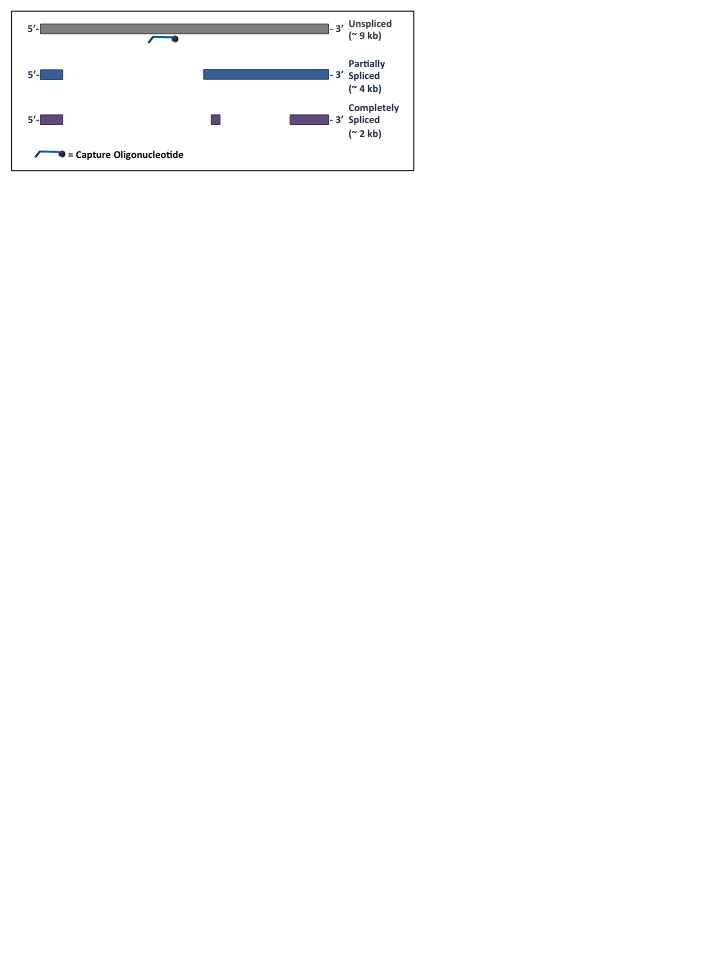


**Figure S1:** The capture oligonucleotide was designed to be complementary to 30 nucleotides of the full length transcript that are not present in the partially spliced and completely spliced variants.


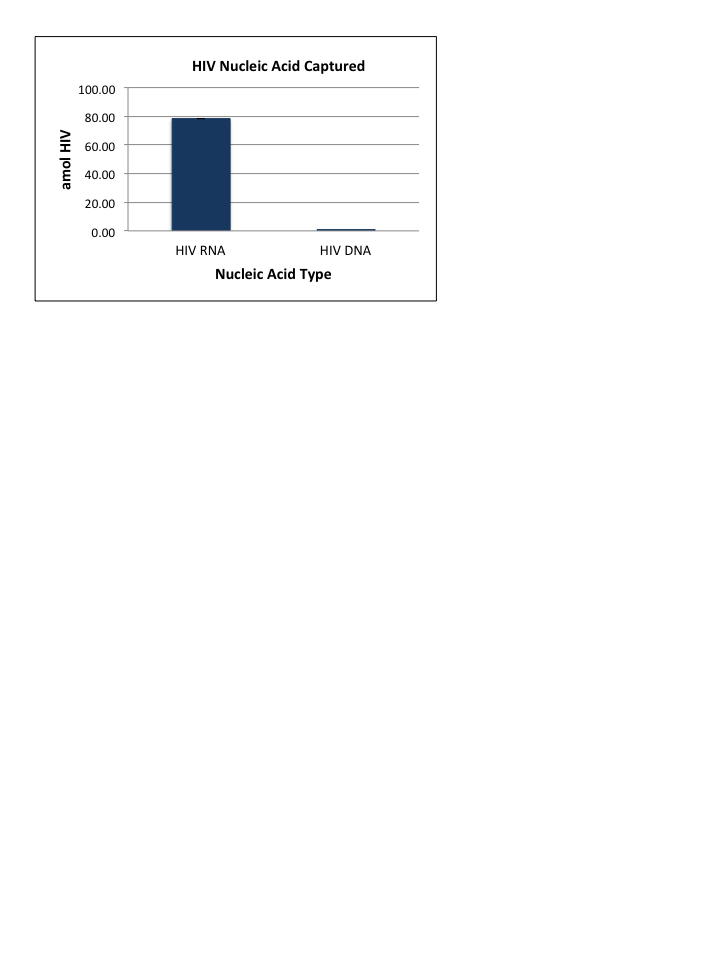


**Figure S2:** Amplification of HIV qPCR region to measure HIV-RNA (sample reverse transcribed then qPCR amplified) and to measure HIV DNA (sample not reverse transcribed then qPCR amplified). Demonstrates that large majority of HIV nucleic acids is in fact RNA, not DNA.


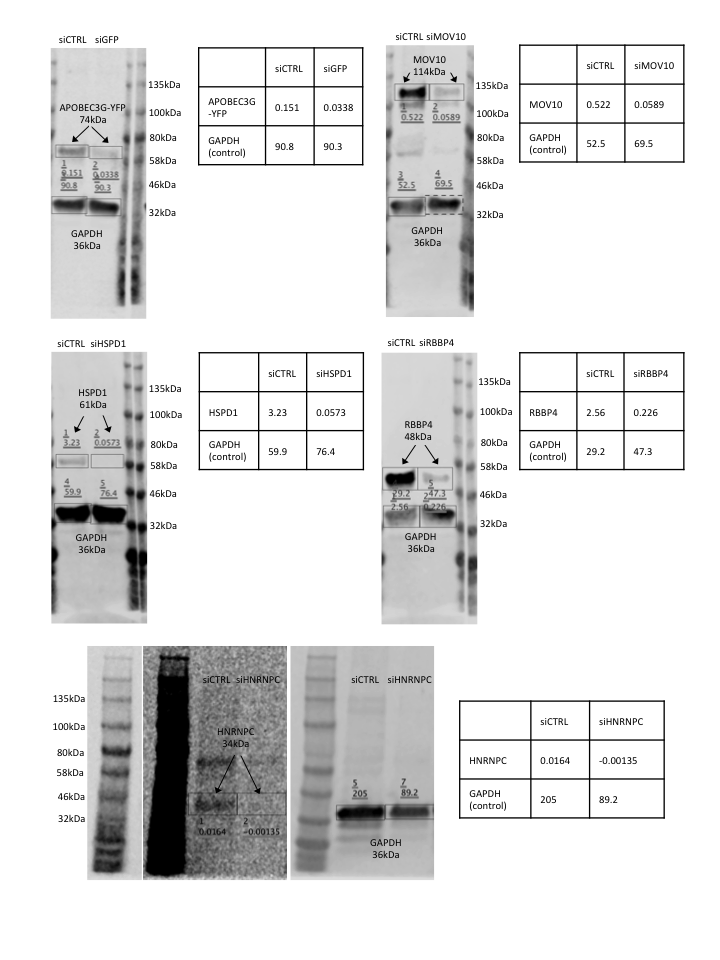


**Figure S3:** Full image of each western blot including the target protein for siRNA knockdown, the loading control (GAPDH) and the ladder with sizes.
